# Dynamics of soil properties and fungal community structure in continuous-cropped alfalfa fields in Northeast China

**DOI:** 10.7717/peerj.7127

**Published:** 2019-06-13

**Authors:** Qin Yao, Yanxia Xu, Xuefeng Liu, Junjie Liu, Xinyu Huang, Weiguang Yang, Zhao Yang, Lan Lan, Jingming Zhou, Guanghua Wang

**Affiliations:** 1 Key Laboratory of Mollisols Agroecology, Northeast Institute of Geography and Agroecology, Chinese Academy of Sciences, Harbin, China; 2 Branch of Animal Husbandry and Veterinary of Heilongjiang Academy of Agricultural Sciences, Qiqihar, China

**Keywords:** Alfalfa, Continuous cropping, Community structure, Illumina MiSeq sequencing

## Abstract

To compensate for the seasonal imbalance between livestock and forage yield in the cold region of Northeast China, alfalfa (*Medicago sativa* L.) continuous cropping has been widely employed in animal husbandry. However, the effects of continuous cropping of alfalfa on soil properties, including physical, chemical and biological properties, are poorly understood. In this study, we investigated the soil properties and fungal community composition of alfalfa fields under continuous cropping for different time periods (i.e., 1, 2, 6, 9, 12, 13 and 35 years). The results showed that soil moisture, total C, total N, NO_3_^−^-N and available K content decreased at less than 10 years of continuous cropping and then increased at more than 10 years of continuous cropping, but soil total P and available P content showed the opposite tendency. The soil fungal community composition determined using Illumina Miseq sequencing showed that continuous cropping increased the fungal alpha diversity and changed the fungal community structure. The relative abundances of *Guehomyces* and *Chaetomium* decreased, but the relative abundances of *Phaeomycocentrospora* and *Paecilomyces* increased with continuous cropping time. In addition, continuous cropping of alfalfa increased the relative abundances of some plant pathogens, such as *Haematonectria haematococca* and *Cyphellophora* sp. Soil total P and available P content were important soil factors affecting the soil fungal community diversity, fungal community structure and the relative abundances of specific fungi in this alfalfa continuous cropping system.

## Introduction

Alfalfa (*Medicago sativa* L.), as an important perennial herbaceous forage legume, is widely grown in many countries (Raiesi, 2007; Su, 2007; Li & Huang, 2008; Bagavathiannan, Gulden & Van Acker, 2011) with a great contribution to the development of agriculture and animal husbandry (Han, Jia & Wang, 2005). In China, alfalfa is cultivated in more than 4 × 10^6^ hectares every year and is mainly planted in the arid and semiarid regions of northern China (Zhang et al., 2016). Northeast China is an ecotone system between agriculture and animal husbandry, and winter in this region is longer than in other parts of China (Chen et al., 2013). Thus, the animal fodder in this area nearly exclusively relies on pasture in summer and on silage in winter (Su, 2007). Alfalfa could eliminate the seasonal imbalance between livestock and forage yield in Northeast China due to its great yield potential, high nutritional value and wide adaptation (Chen et al., 2013). Therefore, to meet forage demand in the winter season and then enhance the productivity of livestock, a large area of alfalfa was planted continuously in Northeast China (Dong et al., 2003).

Previous studies showed that the length of the alfalfa growth phase was related to grass yield. Alfalfa productivity increased within 8 years after establishment in the dryland region of northwestern China and then decreased when alfalfa continuously grew for >8 years (Li & Huang, 2008). In addition, a study in the semiarid Loess Plateau of China recommended that the optimal length of the alfalfa growth phase is 9 years (Jiang et al., 2007). These studies also indicated that the soil quality of alfalfa fields changed with increasing age and is reflected in alfalfa productivity (Jiang et al., 2007; Ren et al., 2011). Soil quality, including physical, chemical and biological properties, can directly or indirectly influence soil productivity and environmental security (Doran & Parkin, 1994). Dong et al. (2016) found that the amounts of soil organic carbon, total nitrogen, total phosphorus and available phosphorus were significantly increased when new alfalfa land was reclaimed from native sandy steppe. A pot experiment also indicated that planting alfalfa significantly increased the contents of soil organic matter, total nitrogen, available nitrogen, available phosphorus and available potassium (Luo et al., 2018). However, a long-term survey showed that the contents of soil organic carbon, total nitrogen, available phosphorus and soil nitrate nitrogen decreased within 10 years of continuous cropping of alfalfa but then increased after alfalfa grew for more than 10 years (Jiang et al., 2007).

Soil microorganisms are important components of soil ecosystems, play a critical role in material cycling (Lupwayi et al., 2004; Bastida et al., 2017), and quickly respond to changes in soil physicochemical properties (Jiang et al., 2007; Xiao et al., 2017). However, studies related to soil microbial characteristics in alfalfa fields have been limited (Beauregard et al., 2010; Zhong et al., 2012; Luo et al., 2018). Jiang et al. (2007) investigated the soil microbial properties under alfalfa continuous cropping fields in the Loess Plateau of China and found that soil microbial biomass and soil basal respiration decreased steadily from 3 years of alfalfa continuous cropping to 9 years but increased from 15 to 25 years. Luo et al. (2018) assessed the influence of alfalfa revegetation on the soil microbial community in an Entisol of East China and found that alfalfa revegetation significantly increased soil microbial diversity (e.g., operational taxonomic units (OTUs) richness and Shannon index) and affected the soil microbial community structures through changes in soil physicochemical properties. In addition to the continuous growth of alfalfa, continuous cropping of soybean caused the gradual transformation of soil from “bacterial type” to “fungal type,” as continuous cropping enhances fungal growth while inhibits bacterial proliferation (Jie, Liu & Cai, 2013). In particular, the abundance of pathogenic fungi, which could influence plant growth and crop yield, was increased with continuous cropping (Guo et al., 2011; Bai et al., 2015).

Therefore, considering the change in soil microorganisms (especially fungi or pathogenic fungi), to support the sustainable development of animal husbandry in Northeast China, it is necessary to reveal the relationships between soil quality and long-term continuous cropping of alfalfa. In this study, soil samples were collected from continuous-cropped alfalfa fields of different cropping times in Northeast China, and the soil properties and fungal communities were investigated. The objectives of this study were (1) to assess the dynamic changes in soil properties and fungal community structures with continuous cropping time and (2) to estimate the comprehensive relationships among soil properties, soil fungal communities and continuous cropping time.

## Materials and Methods

### Study site and experimental design

The research fields were set up in an experimental field of the Heilongjiang Province Institute of Animal Science, which is located in the Fularji district (47°15′N, 123°41′E), Qiqihar, Heilongjiang Province, China. The average annual temperature is 3 °C, and the average annual precipitation is 450 mm in this area. The soil is aeolian sandy soil, the pH value of the soil is 7.4 and the salinity is 0.24%.

Fields with alfalfa continuous cropping for 1, 2, 6, 9, 12, 13 and 35 years were selected for this study, which were encoded ACC1y, ACC2y, ACC6y, ACC9y, ACC12y, ACC13y and ACC35y, respectively. All the treatments were randomly arranged in a large experimental field, and each treatment covered more than 900 m^2^ in area. At the beginning of the established experiment, alfalfa (*Medicago sativa* L.cv. Longmu801) was seeded at a density of 3,000,000 seeds ha^−1^. Chemical compound fertilizer (N 16%, P_2_O_5_ 16%, K_2_O 16%) was annually applied at 280 kg ha^−1^ in each experimental plot in late May. The alfalfa fields were managed with conventional cultivation techniques without grazing. The alfalfa was mowed to the soil surface twice and removed from the field in June and August every year except in the first year when the alfalfa was seeded. At other times of year, the alfalfa grew freely.

### Soil sampling and soil property determination

The soil samples were collected at a soil depth of 0–15 cm on June 25, 2015 when the alfalfa was blooming. Each soil sample was a mixture of more than five individual soil cores collected from an area of 300 m^2^ (one-third of the total area) of each treatment. A total of 21 soil samples were obtained from seven continuous cropping alfalfa fields. The soil samples were sieved through a two mm mesh to thoroughly homogenize them, and the visible plant roots, residues and stones were manually removed. All samples were transferred to the laboratory in an ice-cooled box and divided into two groups: one was placed into a 50 mL centrifuge tube and kept at −80 °C for soil DNA analysis, and the other was dried in the room for determination of soil properties, except for ammonium nitrogen and nitrate nitrogen, which were tested with fresh soil.

For the measurement of soil basic properties, we adopted the methods described in our previous paper (Yao et al., 2017). Briefly, the soil pH was determined using a pH meter in a soil water suspension (1:2.5 w/v). The soil moisture content was measured gravimetrically by drying 15 g of fresh soil to a constant weight in a drying oven at 105 °C for 12 h. The soil total carbon (TC) and total nitrogen (TN) contents were measured using an elemental analyzer (VarioEL III, Elementar, Hanau, Germany) (Jones & Willett, 2006). Soil total phosphorus (TP) digested with H_2_SO_4_-HClO_4_, available phosphorus (AP) extracted with 0.5M NaHCO_3_, and ammonium nitrogen (NH_4_^+^-N) and nitrate nitrogen (NO_3_^−^-N) extracted with 2.0M KCl were assayed using a continuous flow analytical system (SAN^++^, SKALAR, Breda, The Netherlands) (Miranda, Espey & Wink, 2001). Soil total potassium (TK) digested with HNO_3_-HClO_4_-HF and available potassium (AK) extracted with 1.0M CH_3_COONH_4_ were quantified using inductively coupled plasma-atomic emission spectrometry (ICPS-7500; Shimadzu, Kyoto, Japan) (Lu, 1999).

### Soil DNA extraction, PCR amplification and Illumina MiSeq sequencing

Soil DNA was extracted from the frozen soil samples (0.5 g wet weight) using a Fast DNA^®^ Spin Kit for Soil (MP Biomedicals, Santa Ana, CA, USA) according to the manufacturer’s instructions and diluted in DES buffer (DNA Elution Solution-Ultra Pure Water). After DNA extraction, the fungal ITS rRNA was amplified using the primers ITS1F/ITS2R (White et al., 1990), with the forward primer modified with a unique six nt barcode at the 5′ end. PCRs were performed using 25 μL PCR mixture containing 0.5 μL of each primer at 10 μM, 1.0 μL of template DNA (10 ng), and 23 μL of Platinum PCR SuperMix (TransGen Biotech Co. Ltd., Beijing, China). The amplification was performed at 94 °C for 3 min, followed by 35 cycles at 94 °C for 30 s, 55 °C for 30 s, and 72 °C for 30 s, followed by an extension at 72 °C for 10 min (Liu et al., 2015). Each sample was amplified for three technical replicates. The PCR products were checked in a 1.5% agarose gel with the Gold View™ nucleic acid stain (Beijing Solarbio Science & Technology Co. Ltd., Beijing, China) and were then purified using the agarose gel DNA purification kit (Takara, Dalian, China). The amplicons from all samples were normalized to equimolar amounts and were sequenced using the Illumina MiSeq platform at the Shanghai Majorbio Biotechnology Company, Shanghai, China.

### Processing of fungal ITS sequencing data

The raw sequence data obtained from Illumina MiSeq sequencing were processed and analyzed using QIIME Pipeline Version 1.8.0 (http://qiime.org/tutorials/tutorial.html) (Caporaso et al., 2010). Briefly, low-quality sequences with a quality score <20 and shorter than 200 bp in length were removed. Chimeric sequences were detected and eliminated using the Uchime algorithm (Edgar et al., 2011). The remaining high-quality sequences were clustered into OTUs at 97% similarity using USEARCH (Edgar, 2010). The representative sequence of OTUs was aligned using the Python nearest alignment space termination (DeSantis et al., 2006; Caporaso et al., 2010) with a phylogenetic tree built using Fast Tree (Price, Dehal & Arkin, 2009). The taxonomic classification of each representative OTU was assigned using a BLAST comparison against sequences within the GenBank database. In order to analyze the fungal communities at the same sequencing depth, the lowest sequencing number of 29,000 sequences was randomly selected per sample. All sequences have been deposited in GenBank with accession number PRJNA509700.

### Statistical analysis

The Chao1 richness, Shannon index, Simpson index and Phylogenetic diversity were calculated in QIIME and used to compare the fungal alpha diversity among treatments. Significant differences in soil parameters, fungal alpha diversity and the fungal relative abundances of different taxonomic levels among treatments were determined by one-way analysis of variance (ANOVA), and the correlations between fungal relative abundances and soil parameters and continuous cropping years were tested by Pearson’s correlation analysis using SPSS software (version 22.0). Nonmetric multidimensional scaling (NMDS) analysis was performed to compare the fungal beta diversity between treatments, and canonical correspondence analysis (CCA) was conducted to determine which soil parameters were most frequently related to fungal communities. The NMDS and CCA analyses were all conducted using the “vegan” package in the R environment (R v.2.8.1) (R Core Team, 2016). The fungal OTU taxonomic information was uploaded to FUNGuild (http://www.stbates.org/guilds/app.php) for functional prediction (Nguyen et al., 2016).

## Results

### Soil physicochemical properties

The variations in soil physicochemical properties are displayed in Table 1. Overall, soil moisture, TC, TN, NO_3_^−^-N and AK contents decreased with the extending time from 1 to 9 years and then increased from 9 to 35 years in continuous cropping alfalfa soils (Table 1). In contrast, the contents of TP and AP increased and decreased with cropping years in the treatments of less and more than 10 years, respectively. The soil pH value and TK content fluctuated with the cropping year. The ammonium nitrogen content (NH_4_^+^-N) did not significantly change under alfalfa continuous cropping fields.

**Table 1:** Soil physicochemical properties under alfalfa continuous cropping.

Treatment	pH	Moisture (%)	TC^a^ (g kg^−1^)	TN^a^ (g kg^−1^)	TP^a^ (g kg^−1^)	TK^a^ (g kg^−1^)	NH_4_^+^-N (mg kg^−1^)	NO_3_^−^-N (mg kg^−1^)	AP^a^ (mg kg^−1^)	AK^a^ (mg kg^−1^)
ACC1y^b^	8.34 ± 0.03c^c^	15.41 ± 1.62ab	22.52 ± 0.38a	1.20 ± 0.04b	0.58 ± 0.03b	20.02 ± 0.22bc	57.91 ± 3.21a	5.67 ± 0.46a	8.60 ± 0.42c	128.34 ± 5.25bc
ACC2y	8.43 ± 0.16bc	14.41 ± 0.21b	19.66 ± 0.08bc	1.18 ± 0.02b	0.71 ± 0.04a	20.16 ± 0.46bc	60.17 ± 6.28a	4.17 ± 0.11bc	9.35 ± 0.15c	133.06 ± 5.40b
ACC6y	8.40 ± 0.05c	14.10 ± 0.85bc	18.73 ± 0.62cd	1.19 ± 0.05b	0.72 ± 0.02a	21.19 ± 0.76a	56.47 ± 0.96a	3.92 ± 0.12c	18.05 ± 2.17a	122.36 ± 2.68c
ACC9y	8.60 ± 0.03a	12.73 ± 0.29c	17.39 ± 0.79d	0.83 ± 0.03c	0.69 ± 0.05a	21.46 ± 0.34a	57.51 ± 1.05a	3.93 ± 0.17c	16.16 ± 0.75b	103.93 ± 1.63d
ACC12y	8.54 ± 0.02ab	14.76 ± 0.77ab	24.01 ± 1.49a	1.44 ± 0.14a	0.51 ± 0.04c	19.53 ± 0.59c	56.27 ± 4.16a	4.18 ± 0.14bc	5.79 ± 0.50d	128.58 ± 6.03bc
ACC13y	8.55 ± 0.08ab	14.81 ± 0.48ab	19.08 ± 1.26cd	1.34 ± 0.16ab	0.46 ± 0.01c	20.89 ± 0.02ab	55.91 ± 1.29a	5.45 ± 1.77ab	2.92 ± 0.34e	128.05 ± 0.79bc
ACC35y	8.47 ± 0.01abc	16.26 ± 0.55a	20.88 ± 1.01b	1.54 ± 0.18a	0.40 ± 0.02d	20.78 ± 0.65ab	57.13 ± 2.36a	6.03 ± 0.81a	3.11 ± 0.67e	159.13 ± 8.35a

**Notes:**

a TC, TN, TP, TK, AP and AK indicate soil total carbon, total nitrogen, total phosphorus, total potassium, available phosphorus and available potassium, respectively.

b ACC1y, ACC2y, ACC6y, ACC9y, ACC12y, ACC13y and ACC35y represent the treatments of alfalfa continuous cropping for 1, 2, 6, 9, 12, 13 and 35 years, respectively.

c Different letters within the same column indicate significant difference between treatments tested by one-way ANOVA (*P* < 0.05). Values are the means ± SE (*n* = 3).

### Fungal community composition

In total, 792,738 high-quality sequences were obtained from all soil samples, ranging from 29,798 to 44,636 per soil sample (mean = 37,749) (Table 2). When grouped at the 97% similarity level, 1,911 different phylotypes (OTUs) were obtained across all soil samples, with a mean of 653 phylotypes per soil sample.

**Table 2:** Illumina MiSeq sequenced fungal data and fungal community diversity indices (at 97% sequence similarity) based on the ITS rRNA gene.

Treatment	Fungal sequences	Number of phylotype^a^	Chao1 Richness^a^	Shannon index^a^	Simpson index^a^	Phylogenetic diversity^a^	Coverage (%)^a^
ACC1y^b^	36,757 ± 5,250^c^	625 ± 29de^d^	837 ± 21a	4.40 ± 0.05d	0.030 ± 0.003a	255.41 ± 8.76c	99.42
ACC2y	37,751 ± 3,436	603 ± 13e	770 ± 28bc	4.56 ± 0.07c	0.020 ± 0.002b	249.91 ± 4.63c	99.47
ACC6y	41,112 ± 1,377	555 ± 33f	689 ± 34d	4.17 ± 0.06e	0.027 ± 0.005a	216.41 ± 3.31d	99.49
ACC9y	38,180 ± 7,604	655 ± 14cd	749 ± 42c	4.69 ± 0.14bc	0.018 ± 0.000b	259.63 ± 7.93c	99.51
ACC12y	36,652 ± 2,427	686 ± 19bc	814 ± 11ab	4.62 ± 0.08bc	0.022 ± 0.000b	274.23 ± 9.17b	99.49
ACC13y	37,392 ± 5,169	698 ± 5b	860 ± 31a	4.74 ± 0.07ab	0.019 ± 0.000b	283.13 ± 1.32b	99.43
ACC35y	36,402 ± 5,219	746 ± 6a	845 ± 40a	4.84 ± 0.04a	0.019 ± 0.003b	300.31 ± 1.31a	99.43

**Notes:**

a Those data were calculated from 29,000 fungal sequences per soil sample.

b ACC1y, ACC2y, ACC6y, ACC9y, ACC12y, ACC13y and ACC35y represent the treatments of alfalfa continuous cropping for 1, 2, 6, 9, 12, 13 and 35 years, respectively.

c Values are the means ± SE (*n* = 3).

d Different letters within the same column indicate significant difference between treatments tested by one-way ANOVA (*P* < 0.05).

**Figure 1:**
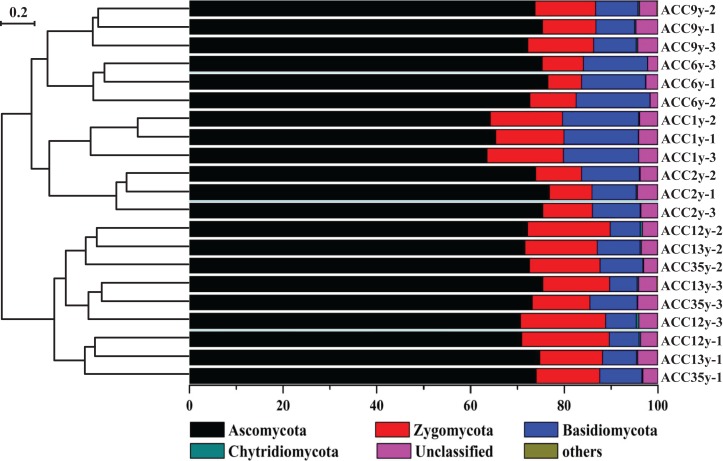
Phylogenetic relationships of fungal communities shown with the relative abundances of different fungal phyla. ACC1y, ACC2y, ACC6y, ACC9y, ACC12y, ACC13y and ACC35y represent the treatments of alfalfa continuous cropping for 1, 2, 6, 9, 12, 13 and 35 years, respectively.

The phyla Ascomycota, Zygomycota and Basidiomycota were dominant fungi with relative abundances ranging from 64.37% to 76.15%, from 8.61% to 17.98% and from 6.47% to 15.45% across all samples, respectively (Fig. 1; Table S1). The relative abundance of Ascomycota was significantly and negatively correlated with TC, whereas it was positively correlated with TK. Basidiomycota was significantly and negatively correlated with pH, whereas it was positively correlated with TP and AP. However, Zygomycota was significantly and positively correlated with TC, whereas it was negatively correlated with TP and AP (Table S2).

At the class level, Sordariomycetes was dominant, with a relative abundance of more than 30% across all samples. In addition, two classes, Tremellomycetes and Dothideomycetes, were less abundant (relative abundance >10% in at least one sample) (Table S3), and they had positive or negative correlations with soil pH, TC, TP and continuous cropping year (Table S2).

More than 60 fungal orders were detected across all samples. Among them, Hypocreales, Mortierellales, Sordariales and Pleosporales were abundant orders, with a relative abundance of more than 5% (Table S4). The relative abundance of the most abundant order, Hypocreales, which belongs to the Sordariomycetes class of Ascomycota, was positively and negatively correlated with cropping year and TP, respectively, while the order Sordariales had the opposite correlations (Tables S2 and S4).

**Figure 2:**
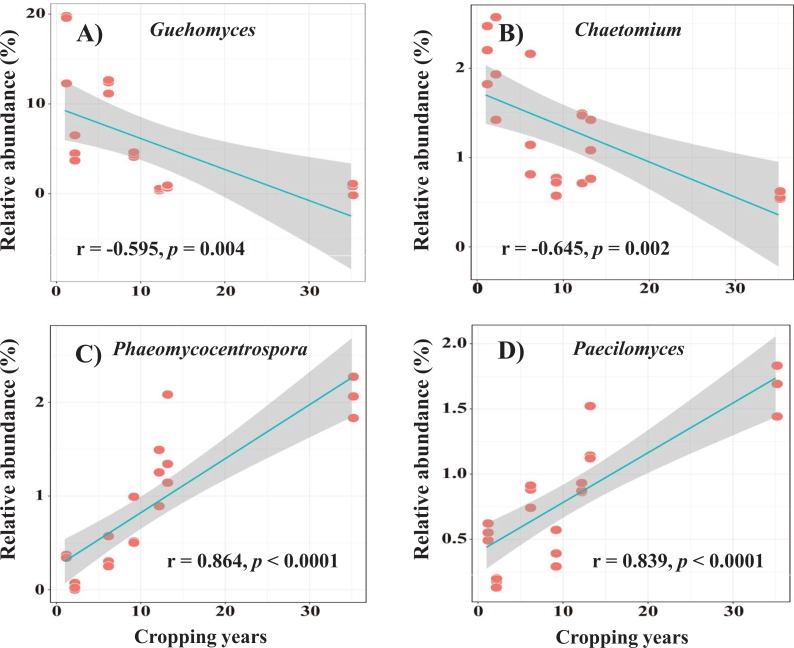
The relationship between relative abundances of dominant fungal genera and alfalfa continuous cropping years. A–D represent the genus *Guehomyces*, *Chaetomium*, *Phaeomycocentrospora* and *Paecilomyces*, respectively.

More than 300 fungal genera were detected across all samples. Among them, 38 abundant fungal genera (relative abundance > 0.3%) accounted for more than 80% of the fungal sequences (Table S5). *Guehomyces* and *Mortierella* were dominant genera, and their relative abundances varied from 0.42% to 17.19% and 8.42% to 16.74%, respectively (Table S5). The relative abundance of *Guehomyces* was positively correlated with soil TP and AP but negatively correlated with soil pH and alfalfa continuous cropping year (*r* = −0.595, *P* = 0.004) (Table S2; Fig. 2A). The relative abundance of *Mortierella* was negatively correlated with soil TP and AP but positively correlated with soil TC (Table S2). In addition, the relative abundances of three less abundant genera, *Chaetomium* (*r* = −0.645, *P* = 0.002), *Phaeomycocentrospora* (*r* = 0.864, *P* < 0.0001) and *Paecilomyces* (*r* = 0.839, *P* < 0.0001), were negatively and positively correlated with alfalfa continuous cropping year, respectively (Table S2; Figs. 2B–2D), and they also had significant correlations with some soil properties, such as soil pH, TN, TP, AP and AK (Table S2).

### Fungal functional groups

**Figure 3:**
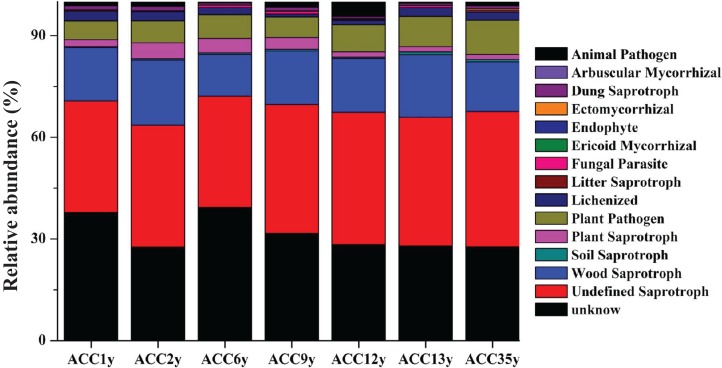
Variations in composition of fungal functional groups inferred by FUNGuild. ACC1y, ACC2y, ACC6y, ACC9y, ACC12y, ACC13y and ACC35y represent the treatments of alfalfa continuous cropping for 1, 2, 6, 9, 12, 13 and 35 years, respectively.

Among the 1,911 OTUs detected in this study, 866 OTUs (45.32% of the total OTUs) were annotated to 14 functional groups based on the FUNGuild database (Fig. 3; Table S6). The relative abundances of plant pathogens and plant saprotrophs were significantly different between the alfalfa continuous cropping treatments of less than and more than 10 years, except for some treatments (Table S6). In addition, the highest abundance of fungal parasites appeared in ACC9y (1.12%) (Table S6).

**Figure 4:**
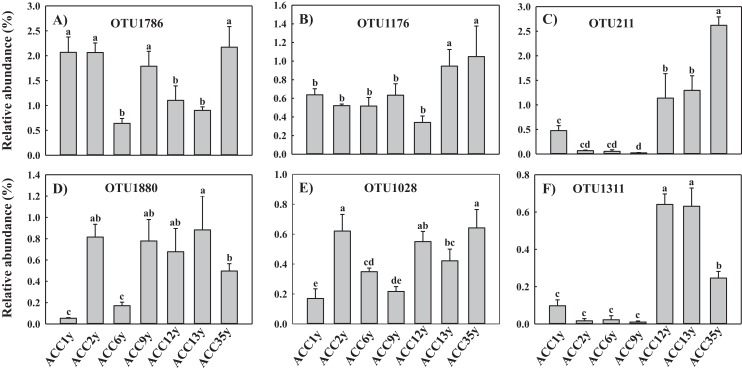
Effect of alfalfa continuous cropping on the relative abundance of dominant plant pathogens at the OTUs level. Different letters above columns indicate significant difference between treatments tested by one-way ANOVA (*P* < 0.05). Error bar show mean ± SE (*n* = 3). ACC1y, ACC2y, ACC6y, ACC9y, ACC12y, ACC13y and ACC35y represent the treatments of alfalfa continuous cropping for 1, 2, 6, 9, 12, 13 and 35 years, respectively. A–F represent OTU1786, OTU1176, OTU211, OTU1880, OTU1028 and OTU1311, respectively.

A total of 84 OTUs were assigned as the functional group of plant pathogen (Table S6). Among them, the top six OTUs belonged to the phylum Ascomycota, with relative abundances ranging from 0.01% to 2.62% (Fig. 4; Table S7). Of these, the relative abundances of OTU211 (*Haematonectria haematococca*) and OTU1311 (*Cyphellophora* sp.) under continuous cropping alfalfa for more than 10 years were significantly higher than those under continuous cropping for less than 10 years (Figs. 4C and 4F). Their abundances were positively correlated with soil TN and continuous cropping year and negatively correlated with soil TP and AP (Table S7). The relative abundances of OTU1176 (*Fusarium incarnatum*) in ACC13y and ACC35y were significantly higher than that in other treatments (Fig. 4B). OTU1176 abundance had a negative correlation with soil TP and a positive correlation with soil NO_3_^−^-N, AK and alfalfa continuous cropping year (Table S7). However, the relative abundances of the other three dominant OTUs (OTU1786, OTU1880 and OTU1028) did not change regularly with continuous cropping year (Figs. 4A, 4D and 4E), but they had significant correlations with some soil properties, such as soil pH, TN and AK (Table S7).

### α-diversity pattern of soil fungal communities

**Figure 5:**
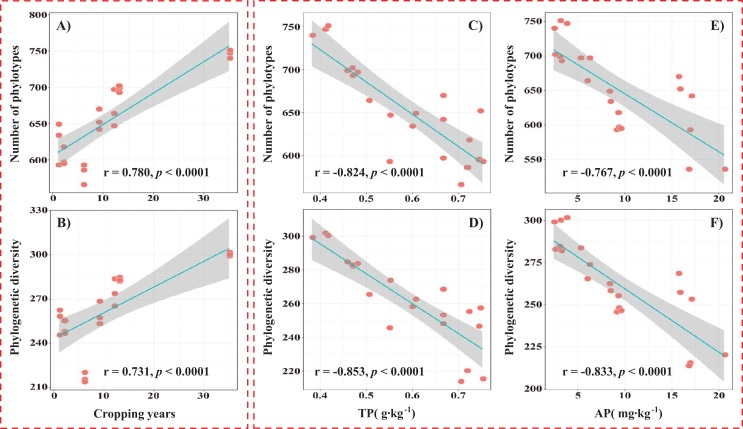
The relationship between soil fungal phylotype richness or phylogenetic diversity and alfalfa continuous cropping year (A and B), soil TP (C and D) or AP (E and F).

To compare the α-diversity of soil fungal communities, the same survey effort level of 29,000 sequences was randomly selected from each sample. The coverage values of all the samples were more than 99% (Table 2), indicating that the current sequencing depth was sufficient to capture the fungal diversity. The number of phylotypes ranged from 555 to 746, and phylogenetic diversity ranged from 216 to 300 across all soil samples (Table 2). Pairwise analysis showed that both phylotype richness (*r* = 0.780, *P* < 0.0001) and phylogenetic diversity (*r* = 0.731, *P* < 0.0001) of the soil fungal community increased with the cropping years (Figs. 5A and 5B). Among soil properties, the soil TP and AP contents were significantly and negatively correlated with both the phylotype richness and phylogenetic diversity (Figs. 5C–5F). In addition, other α-diversity indices, Chao1 richness, Shannon index and Simpson index, also differed among different treatments (Table 2).

### β-diversity pattern of soil fungal communities

**Figure 6:**
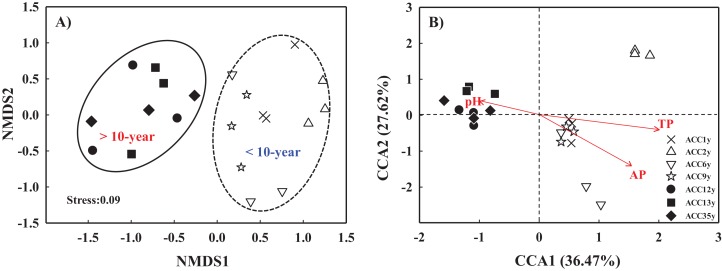
Nonmetric multidimensional scaling (NMDS) plot of soil fungal communities for different continuous cropping treatments (A) and canonical correspondence analysis (CCA) of fungal community changes with soil variables (B). ACC1y, ACC2y, ACC6y, ACC9y, ACC12y, ACC13y and ACC35y represent the treatments of alfalfa continuous cropping for 1, 2, 6, 9, 12, 13 and 35 years, respectively.

Based on Bray–Curtis distance dissimilarity, the β-diversity of the fungal communities was evaluated with NMDS analysis (Fig. 6A). The NMDS plot exhibited the best separation of fungal communities of alfalfa continuous cropping for less than 10 years from those for more than 10 years, and thus all samples were separated into two major groups (Fig. 6A). A similar result was also found by the clustering analysis (Fig. 1). Within each group, the fungal communities under alfalfa continuous cropping for less than or more than 10 years were not well separated.

A CCA plot was employed to identify the major soil variables that affected the fungal community structure. Based on the results of the Mantel test (Table S8), soil parameters significantly correlated with the fungal community structure were selected for the CCA analysis (Fig. 6B). Of all the tested soil variables, soil TP (*r* = 0.463, *P* = 0.001) had the longest arrow along the CCA1 axis, which indicated that soil TP was the most important soil parameter in shifting the soil fungal communities. The second most important soil factor was soil AP (*r* = 0.399, *P* = 0.001) (Fig. 6B; Table S8). In addition, soil pH (*r* = 0.175, *P* = 0.020) also made an important contribution to the change in soil fungal community structure (Fig. 6B; Table S8).

## Discussion

### Variation in soil properties under alfalfa continuous cropping

Soil properties are influenced by long-term planting or continuous cropping (Fu et al., 2017; Chen et al., 2018). In this study, alfalfa continuous cropping resulted in a change in soil properties in the arid Songnen Plain of Northeast China (Table 1). The soil moisture, TC, TN and NO_3_^−^-N contents first decreased and then increased with the extension of cultivation years. This result is consistent with the report of Jiang et al. (2007), who found that these soil parameters under alfalfa continuous cropping fields, which were influenced by nutrient uptake and litter input to soil, decreased with the greater amount of dry alfalfa grass removed from the field from 3 to 9 years, while the soil parameters increased from 15 to 25 years in the semiarid Loess Plateau of Northwest China. In addition, the contents of soil TP and AP in our study increased with planting years under short-term alfalfa continuous cropping conditions (<10 years) (Table 1). This result was basically consistent with the finding of Dong et al. (2016), who stated that the soil TP and AP contents in alfalfa soils increased significantly after applying fertilizer in a short-term period in the Heihe River Basin of an arid region of Northwest China. In addition, the soil AP content in the treatments of alfalfa continuous cropping for less than 10 years was significantly higher than that in the treatments of continuous cropping for more than 10 years. This result was also found in a long-term investigation by Fan et al. (2011), who reported that after alfalfa grew for more than 10 years, harvesting of the alfalfa with higher density removed more phosphorus and returned less plant residue to soil, resulting in a decrease in soil AP content in the Loess Plateau of northern China. In short, alfalfa continuous cropping led to changes in soil properties, and 10 years may be the optimal length for the alfalfa continuous cropping system in view of soil quality.

### Variation in soil fungal community composition under alfalfa continuous cropping

In our study, we found that Ascomycota, Zygomycota and Basidiomycota were the dominant phyla, while Chytridiomycota was a minor phylum across all soil samples (Fig. 1; Table S1). A similar result was also reported in a *Panax notoginseng* continuous cropping field (Tan et al., 2017), suggesting that Ascomycota, Zygomycota and Basidiomycota are abundant fungal phyla in continuous cropping systems. In addition, continuous cropping significantly elevated and reduced the relative abundances of Ascomycota and Basidiomycota, respectively, especially compared to their abundances in ACC1y (Table S1). This result is basically consistent with the report of Luo et al. (2018), who found that planting alfalfa significantly changed the soil properties (e.g., pH, TC, TP and AP) for microbial growth and subsequently increased the relative abundance of Ascomycota and reduced that of Basidiomycota in alfalfa fields in eastern China.

At the genus level, the relative abundance of *Chaetomium* decreased with the extension of continuous cropping time (Fig. 2B). *Chaetomium* belongs to Ascomycota and has the ability to degrade cellulose with efficient cellobiose dehydrogenase activity (Harreither et al., 2011; Abdelkader & Hamed, 2013). Moreover, *Chaetomium* was reported as a potential biocontrol agent that can resist some soil-borne pathogens by producing antifungal compounds (Huang et al., 2016). Meng et al. (2018) found that the abundance of the pathogen *F. oxysporum* was negatively correlated with the relative abundance of the antagonist *Chaetomium*. The decrease in the relative abundance of *Chaetomium* in our study indicated that alfalfa continuous cropping may suppress the growth of antagonistic fungi. In addition, we observed that the relative abundance of *Paecilomyces* increased with prolonged alfalfa continuous cropping time (Fig. 2D) and had a positive correlation with soil moisture (Table S2). This result was consistent with that in a previous study by Mackie et al. (1999), who stated that dry conditions could restrict the expression of fungal disease, in view of the many species in the genus *Paecilomyces* that are pathogens (Notarte et al., 2018; Piekarska, Trusz & Szczesniak, 2018). Meanwhile, some species of this genus were demonstrated as biocontrol agents, such as *Paecilomyces lilacinus* (Wei et al., 2015; Abd-Elgawad & Askary, 2018) and *Paecilomyces fumosoroseus* (Ruiu, 2018). Furthermore, some species of *Paecilomyces* could cause food contamination and spoilage (Ismail, 2001) as well as play an important role in soil carbon turnover (Kluczek-Turpeinen et al., 2007). We also found that the relative abundance of *Paecilomyces* in alfalfa soils was positively correlated with soil TN and AK content and negatively correlated with soil TP and AP content (Table S2). These soil properties were significantly influenced by alfalfa continuous cropping (Table 1). In this case, the genus *Paecilomyces* of Ascomycetes has a complex role in the environment, and our future study should be deeply focused on its role in the alfalfa continuous cropping system.

At the final phylogenetic resolution level, 84 OTUs were assigned as plant pathogens by FUNGuild analysis, and the relative abundance of plant pathogens increased with the extension of the alfalfa continuous cropping time (Fig. 3; Table S6). This result was consistent with the findings of Li et al. (2014), who stated that continuous cropping increased the abundance of pathogenic fungi in peanut soil. Similarly, the abundances of soil-borne pathogens were increased in continuous cropping fields of cucumber (Feng et al., 2016), tomato (Fu et al., 2017) and potato (Liu et al., 2014). These findings suggested that the environmental conditions under long-term continuous cropping were likely to be prone to pathogen proliferation. In particular, we found that the relative abundance of OTU211, which was assigned to *H. haematococca* of Ascomycota phylum, was significantly increased when the alfalfa continuous cropping time was longer than 10 years (Fig. 4C). *H. haematococca* is a plant pathogen, and its teleomorph is *F. solani*, which is virulent and causes alfalfa root rot (Li, Li & Meng, 2005; Cao et al., 2008; Kong et al., 2018). Moreover, the relative abundance of OTU211 (*H. haematococca*) had significant correlations with soil moisture, TN, TP, AP and AK (Table S7), indicating that alfalfa continuous cropping may cause the occurrence of root rot that influenced the alfalfa growth indirectly through the changes in soil edaphic properties. In addition, the relative abundance of OTU1311, which was assigned to *Cyphellophora* sp., was also significantly higher in the treatments with continuous cropping for more than 10 years than that in the treatments of less than 10 years (Fig. 4F). Some species of the fungal genus *Cyphellophora* are potential pathogens (Decock et al., 2003; Ma et al., 2018), and the relative abundance of OTU1311 was correlated significantly with some soil properties, such as soil TC, TN, TP and AP (Table S7), suggesting that continuous cropping may promote the proliferation of pathogens (Guo et al., 2011; Ranzi et al., 2017).

### Variation in soil fungal diversity under alfalfa continuous cropping

Previous studies showed that the taxonomic richness and diversity of soil microorganisms were strongly influenced by continuous cropping (Jie, Liu & Cai, 2013; Li et al., 2014; Bainard et al., 2017; Tan et al., 2017). In our study, we observed that the number of phylotypes and the phylogenetic diversity were significantly increased with continuous cropping time (Table 2; Figs. 5A and 5B), which was consistent with a previous report in cropping wheat field soils (Bainard et al., 2017). Similarly, a study in soybean fields of Northeast China indicated that continuous cropping increased the fungal community diversity (Bai et al., 2015). However, soil fungal community diversity in a long-term experiment of monoculture soybean detected by DGGE pattern was not significantly influenced by continuous cropping (Li et al., 2010). The discrepancy may be mainly due to the sensitivity and limitations of the molecular methods (Bai et al., 2015). In addition, the soil fungal community diversity in our study was negatively correlated with soil TP and AP contents (Figs. 5C–5F), which were significantly influenced by continuous cropping (Table 2), indicating that continuous cropping could indirectly affect the soil fungal community diversity by changing the soil properties.

### Variation in soil fungal community structure under alfalfa continuous cropping

In this study, alfalfa continuous cropping significantly changed the soil fungal community structure (Fig. 6A). This result agreed with the findings of Bai et al. (2015), who stated that the fungal community structure in soybean soils was influenced by continuous cropping using 454 high-throughput sequencing analysis. Similarly, Song et al. (2018) found that changes in soil nutrients and pH caused by the continuous cropping of *Coptis chinensis* affected fungal survival and growth, thereby significantly altered fungal community composition. In addition, the communities under continuous cropping for more than 10 years were obviously different from those of less than 10 years (Fig. 6A), indicating that 10 years may be a cut-off point in the variation of soil fungal communities under alfalfa continuous cropping. Moreover, the structure of the microbial community could also be altered by soil properties (Yao, Jiao & Wu, 2006; Pulleman et al., 2012; Tan et al., 2017; Chen et al., 2018). In this study, the CCA plot showed that soil pH, TP and AP were the dominant factors in shifting the soil fungal community structure in continuous cropping alfalfa fields (Fig. 6B). Similar results were also reported by Song et al. (2018), who stated that some soil properties, such as soil pH and AP, displayed significant effects on the fungal community composition. These soil properties in our study were markedly influenced by continuous cropping (Table 1), indicating that continuous cropping altered soil characteristics and then changed soil fungal community structure (Tan et al., 2017).

## Conclusions

In summary, long-term continuous cropping of alfalfa altered the soil properties and soil fungal community structure and increased the soil fungal alpha diversity. In particular, alfalfa continuous cropping influenced the relative abundances of some plant pathogens, such as *H. haematococca* and *Cyphellophora* sp. The soil TP and AP contents, which were significantly affected by alfalfa continuous cropping, were not only negatively correlated with soil fungal community diversity but also significantly correlated with soil fungal community structure and the relative abundance of specific fungi at the different classification levels. In the end, we suspected that the optimal length of alfalfa continuous cropping may be approximately 10 years according to the variation in soil basic properties and soil fungal community composition, and the further isolation and identification of plant pathogens detected in this study will be required in future research.

## Supplemental Information

10.7717/peerj.7127/supp-1 Supplemental Information 1 Relative abundances (%) of fungal phylum across all soil samples.

10.7717/peerj.7127/supp-2 Supplemental Information 2 Pearson’s correlation of dominant phyla (relative abundance > 0.1%), classes (> 0.03%), orders (> 0.1%) and genera (0.3%) with environmental variables.

10.7717/peerj.7127/supp-3 Supplemental Information 3 Relative abundances (%) of the dominant fungal classes of all soil samples (> 0.03% at least in one treatment).

10.7717/peerj.7127/supp-4 Supplemental Information 4 Relative abundances (%) of the dominant fungal orders of all soil samples (> 0.1% at least in one treatment).

10.7717/peerj.7127/supp-5 Supplemental Information 5 Relative abundances (%) of the dominant fungal genus of all soil samples (> 0.3% at least in one treatment).

10.7717/peerj.7127/supp-6 Supplemental Information 6 Relative abundances (%) of different fungal functional guilds across all samples.

10.7717/peerj.7127/supp-7 Supplemental Information 7 Relative abundance (%) and taxa information of dominant OTUs belonged to plant pathogen (a) and their correlations with soil environmental variables (b).

10.7717/peerj.7127/supp-8 Supplemental Information 8 Mantel test results for the correlation between fungal community composition and environmental variables.

## Additional Information and Declarations

### Data Availability

The following information was supplied regarding data availability: Data is available at GenBank, accession number: PRJNA509700.
