# Supplementary material for: Dynamics of soil properties and fungal community structure in continuous-cropped alfalfa fields in Northeast China
Source: PeerJ. 2019 Jun 13;7:e7127. doi: 10.7717/peerj.7127 (PMC6571135; doi:10.7717/peerj.7127)
Supplement: Supplemental Information 1 [file peerj-07-7127-s001.docx]

**Table S1** Relative abundance (%) of fungal phylum across all soil samples

| Phylum | ACC1y^a^ | ACC2y | ACC6y | ACC9y | ACC12y | ACC13y | ACC35y |
| --- | --- | --- | --- | --- | --- | --- | --- |
| Ascomycota | 64.37±0.94c^b^ | 74.73±1.85a | 76.15±3.29a | 73.77±1.61a | 69.57±1.17b | 75.29±3.95a | 73.25±0.74ab |
| Zygomycota | 15.36±0.92b | 9.16±0.61d | 8.61±1.37d | 12.83±1.34c | 17.98±0.88a | 14.37±1.04bc | 13.68±1.39bc |
| Basidiomycota | 15.45±1.18a | 10.69±1.51b | 14.4±1.21a | 8.67±0.39bc | 6.47±0.06d | 7.51±1.74cd | 9.31±0.71bc |
| Chytridiomycota | 0.12±0.08de | 0.19±0.06bcd | 0.07±0.02e | 0.30±0.04b | 0.46±0.10a | 0.29±0.03bc | 0.18±0.05cde |
| Unclassified | 3.56±0.29ab | 3.62±0.58ab | 2.05±0.48c | 4.17±0.43a | 3.20±0.28b | 3.81±0.38ab | 3.41±0.73ab |
| others | 0.08±0.03ab | 0.11±0.03ab | 0.03±0.01b | 0.10±0.07ab | 0.11±0.05ab | 0.14±0.07a | 0.07±0.03ab |

^a^ ACC1y, ACC2y, ACC6y, ACC9y, ACC12y, ACC13y and ACC35y represent the treatments of alfalfa continuous cropping for 1, 2, 6, 9, 12, 13 and 35 years, respectively.

^b^ Different letters within the same row indicate significant difference between treatments tested by One-Way ANOVA (*P* < 0.05). Values are the means ± SE (n = 3).
