# Supplementary material for: Dynamics of soil properties and fungal community structure in continuous-cropped alfalfa fields in Northeast China
Source: PeerJ. 2019 Jun 13;7:e7127. doi: 10.7717/peerj.7127 (PMC6571135; doi:10.7717/peerj.7127)
Supplement: Supplemental Information 2 [file peerj-07-7127-s002.docx]

**Table S2**. Pearson’s correlation of dominant phyla (relative abundance > 0.1%), classes (> 0.03%), orders (> 0.1%) and genera (0.3%) with environmental variables

| **Phylum** | pH | Mositure | TC | TN | TP | TK | NH_4_^+^–N | NO_3_^–^–N | AP | AK | Year |
| --- | --- | --- | --- | --- | --- | --- | --- | --- | --- | --- | --- |
| Ascomycota | 0.316 | –0.325 | –0.631^**^ | –0.014 | 0.223 | 0.561^**^ | 0.090 | –0.303 | 0.176 | –0.054 | 0.193 |
| Zygomycota | 0.244 | 0.249 | 0.620^**^ | 0.303 | –0.669^**^ | –0.391 | –0.226 | 0.258 | –0.556^**^ | 0.096 | 0.239 |
| Basidiomycota | –0.759^**^ | 0.098 | –0.037 | –0.225 | 0.443^*^ | 0.042 | 0.116 | 0.053 | 0.466^*^ | –0.008 | –0.432 |
| Chytridiomycota | 0.637^**^ | –0.189 | 0.338 | 0.119 | –0.279 | –0.316 | –0.115 | –0.169 | –0.328 | –0.207 | 0.121 |
| **Class** |  |  |  |  |  |  |  |  |  |  |  |
| Sordariomycetes | 0.415 | –0.137 | –0.011 | 0.003 | 0.085 | –0.320 | 0.291 | –0.142 | –0.156 | 0.047 | –0.025 |
| Incertae sedis | 0.285 | 0.142 | 0.452^*^ | 0.096 | –0.540^*^ | –0.286 | –0.075 | 0.270 | –0.383 | 0.029 | 0.215 |
| Dothideomycetes | –0.102 | –0.368 | –0.586^**^ | –0.188 | 0.428 | 0.455^*^ | –0.080 | –0.265 | 0.372 | –0.294 | –0.326 |
| Eurotiomycetes | 0.574^**^ | –0.092 | –0.042 | 0.226 | –0.412 | 0.329 | –0.047 | 0.097 | –0.248 | 0.034 | 0.461^*^ |
| Leotiomycetes | 0.418 | –0.568^**^ | –0.833^**^ | –0.706^**^ | 0.366 | 0.623^**^ | 0.174 | –0.163 | 0.412 | –0.552^**^ | –0.155 |
| Agaricomycetes | 0.246 | 0.544^*^ | 0.284 | 0.597^**^ | –0.727^**^ | –0.322 | –0.138 | 0.376 | –0.796^**^ | 0.650^**^ | 0.681^**^ |
| Tremellomycetes | –0.638^**^ | –0.227 | –0.174 | –0.418 | 0.644^**^ | 0.163 | 0.088 | –0.140 | 0.678^**^ | –0.302 | –0.612^**^ |
| Pezizomycetes | 0.406 | –0.389 | –0.449^*^ | –0.583^**^ | 0.167 | 0.527^*^ | 0.108 | –0.125 | 0.352 | –0.364 | 0.158 |
| Chytridiomycetes | 0.397 | –0.059 | 0.347 | 0.227 | –0.241 | –0.505^*^ | –0.184 | –0.053 | –0.373 | 0.045 | 0.196 |
| Wallemiomycetes | 0.284 | 0.243 | 0.096 | 0.241 | –0.624^**^ | –0.169 | –0.282 | 0.003 | –.535^*^ | 0.276 | 0.582^**^ |
| Microbotryomycetes | –0.216 | 0.313 | 0.016 | –0.047 | –0.315 | 0.102 | –0.081 | 0.350 | –0.204 | 0.160 | 0.129 |
| Orbiliomycetes | 0.314 | 0.124 | 0.217 | 0.248 | –0.326 | –0.104 | –0.228 | 0.268 | –0.306 | –0.213 | –0.114 |
| Glomeromycetes | 0.272 | 0.022 | 0.124 | 0.203 | –0.293 | –0.118 | –0.092 | –0.105 | –0.447^*^ | –0.070 | 0.013 |
| **Order** |  |  |  |  |  |  |  |  |  |  |  |
| Hypocreales | 0.541^*^ | 0.117 | 0.142 | 0.505^*^ | –0.461^*^ | –0.065 | –0.159 | 0.001 | –0.400 | 0.252 | 0.518^*^ |
| Mortierellales | 0.269 | 0.162 | 0.513^*^ | 0.200 | –0.614^**^ | –0.302 | –0.144 | 0.225 | –0.469^*^ | 0.021 | 0.221 |
| Sordariales | –0.402 | –0.068 | 0.034 | –0.311 | 0.480^*^ | –0.269 | 0.523^*^ | 0.012 | 0.182 | –0.037 | –0.505^*^ |
| Pleosporales | –0.075 | –0.371 | –0.627^**^ | –0.182 | 0.395 | 0.477^*^ | –0.106 | –0.290 | 0.353 | –0.288 | –0.279 |
| Thelebolales | 0.232 | –0.726^**^ | –0.702^**^ | –0.851^**^ | 0.756^**^ | 0.514^*^ | 0.180 | –0.483^*^ | 0.804^**^ | –0.745^**^ | –.445^*^ |
| Eurotiales | 0.440^*^ | 0.124 | 0.019 | 0.394 | –0.460^*^ | 0.378 | –0.135 | 0.322 | –0.260 | 0.218 | 0.624^**^ |
| Tremellales | 0.278 | –0.079 | 0.004 | –0.102 | –0.315 | 0.059 | 0.274 | 0.290 | –0.322 | 0.137 | 0.309 |
| Russulales | 0.395 | 0.164 | 0.479^*^ | 0.595^**^ | –0.562^**^ | –0.282 | –0.175 | 0.115 | –0.526^*^ | 0.192 | 0.285 |
| Incertae sedis | 0.094 | –0.053 | –0.063 | –0.014 | –0.035 | –0.167 | 0.070 | –0.027 | –0.224 | 0.048 | 0.015 |
| Agaricales | 0.262 | 0.547^*^ | 0.634^**^ | 0.647^**^ | –0.726^**^ | –0.379 | –0.297 | 0.182 | –0.684^**^ | 0.476^*^ | 0.597^**^ |
| Microascales | 0.268 | 0.044 | 0.339 | –0.065 | –0.082 | –0.365 | 0.360 | –0.002 | –0.233 | 0.138 | 0.068 |
| Pezizales | 0.525^*^ | –0.437^*^ | –0.531^*^ | –0.528^*^ | 0.160 | 0.554^**^ | 0.109 | –0.219 | 0.345 | –0.340 | 0.259 |
| Xylariales | 0.355 | 0.260 | 0.417 | 0.535^*^ | –0.650^**^ | –0.306 | 0.029 | 0.130 | –0.707^**^ | 0.401 | 0.444^*^ |
| Chaetothyriales | 0.481^*^ | 0.304 | 0.034 | 0.425 | –0.765^**^ | 0.103 | –0.120 | 0.463^*^ | –0.688^**^ | 0.319 | 0.550^**^ |
| Cystofilobasidiales | –0.631^**^ | –0.203 | –0.191 | –0.399 | 0.689^**^ | 0.172 | 0.035 | –0.211 | 0.733^**^ | –0.324 | –0.612^**^ |
| Helotiales | 0.321 | –0.279 | –0.600^**^ | –0.420 | 0.139 | 0.452^*^ | 0.326 | –0.055 | –0.040 | –0.286 | –0.222 |
| Capnodiales | 0.137 | –0.118 | –0.315 | –0.203 | 0.172 | 0.005 | 0.374 | 0.064 | –0.182 | –0.013 | –0.210 |
| Onygenales | 0.374 | –0.609^**^ | –0.111 | –0.356 | 0.292 | 0.152 | 0.025 | –0.612^**^ | 0.483^*^ | –0.513^*^ | –0.122 |
| Cantharellales | 0.053 | 0.066 | –0.334 | 0.137 | –0.103 | 0.140 | 0.089 | 0.033 | –0.376 | 0.183 | –0.001 |
| Kickxellales | 0.544^*^ | –0.218 | 0.195 | 0.083 | –0.288 | –0.250 | –0.204 | –0.253 | –0.212 | –0.207 | 0.141 |
| Rhizophlyctidales | –0.003 | –0.295 | –0.284 | –0.259 | 0.420 | –0.051 | 0.219 | –0.400 | 0.106 | –0.118 | –0.355 |
| Basidiobolales | 0.171 | 0.101 | 0.191 | 0.061 | –0.045 | –0.302 | 0.088 | 0.043 | –0.142 | 0.290 | 0.360 |
| Coniochaetales | –0.045 | –0.381 | –0.738^**^ | –0.352 | 0.574^**^ | 0.513^*^ | 0.191 | –0.236 | 0.429 | –0.219 | –0.274 |
| Spizellomycetales | 0.340 | –0.273 | –0.197 | –0.163 | 0.002 | –0.084 | –0.137 | –0.203 | –0.236 | –0.226 | –0.182 |
| Olpidiales | –0.058 | 0.564^**^ | 0.730^**^ | 0.586^**^ | –0.537^*^ | –0.438^*^ | –0.012 | 0.316 | –0.452^*^ | 0.495^*^ | 0.487^*^ |
| **Genus** |  |  |  |  |  |  |  |  |  |  |  |
| *Guehomyces* | –0.655^**^ | 0.050 | 0.030 | –0.320 | 0.475^*^ | –0.011 | 0.023 | –0.014 | 0.497^*^ | –0.251 | –0.595^**^ |
| Ascomycota_Unclassified | –0.602^**^ | 0.440^*^ | 0.283 | 0.025 | 0.121 | –0.272 | –0.012 | 0.186 | 0.160 | 0.054 | –0.282 |
| *Mortierella* | 0.269 | 0.163 | 0.515^*^ | 0.202 | –0.614^**^ | –0.303 | –0.147 | 0.225 | –0.470^*^ | 0.022 | 0.222 |
| *Leptosphaerulina* | –0.333 | –0.172 | –0.310 | –0.063 | 0.511^*^ | 0.256 | 0.051 | –0.305 | 0.486^*^ | –0.181 | –0.419 |
| unidentified | 0.032 | –0.349 | 0.239 | –0.418 | 0.368 | –0.366 | 0.195 | –0.200 | 0.123 | –0.353 | –0.551^**^ |
| Nectriaceae_Unclassified | –0.029 | –0.150 | –0.106 | 0.088 | 0.123 | 0.379 | –0.327 | –0.346 | 0.403 | –0.163 | 0.078 |
| *Gibberella* | –0.047 | 0.164 | –0.073 | 0.018 | –0.267 | 0.042 | 0.220 | 0.466^*^ | –0.261 | 0.378 | 0.420 |
| Fungi_Unclassified | 0.551^**^ | –0.175 | –0.149 | –0.139 | –0.133 | 0.135 | 0.430 | 0.183 | –0.304 | –0.075 | 0.107 |
| *Cryptococcus* | 0.276 | –0.080 | –0.001 | –0.107 | –0.311 | 0.064 | 0.276 | 0.288 | –0.319 | 0.134 | 0.306 |
| Chaetomiaceae_Unclassified | –0.220 | 0.033 | 0.156 | –0.389 | 0.249 | –0.320 | 0.294 | 0.090 | 0.017 | –0.271 | –0.604^**^ |
| *Chaetomium* | –0.513^*^ | 0.127 | 0.241 | –0.125 | 0.330 | –0.529^*^ | 0.173 | –0.067 | 0.050 | –0.109 | –0.645^**^ |
| *Doratomyces* | –0.233 | 0.480^*^ | 0.477^*^ | 0.260 | –0.534^*^ | –0.302 | –0.065 | 0.521^*^ | –0.430 | 0.371 | 0.194 |
| *Cladosporium* | –0.043 | 0.006 | 0.018 | 0.007 | 0.147 | –0.230 | 0.342 | 0.099 | –0.186 | 0.125 | –0.207 |
| *Fusarium* | 0.006 | –0.056 | –0.073 | 0.066 | 0.249 | –0.129 | 0.446^*^ | –0.165 | –0.095 | 0.310 | 0.001 |
| *Penicillium* | 0.470^*^ | 0.029 | 0.390 | 0.194 | –0.278 | –0.220 | –0.008 | –0.161 | –0.275 | –0.071 | 0.208 |
| *Alternaria* | –0.403 | 0.303 | 0.166 | 0.225 | –0.120 | –0.409 | 0.062 | 0.416 | –0.398 | 0.402 | –0.014 |
| Sordariomycetes_Unclassified | –0.046 | –0.155 | –0.447^*^ | –0.124 | 0.204 | 0.535^*^ | –0.151 | –0.153 | 0.517^*^ | 0.029 | 0.364 |
| Sporormiaceae_Unclassified | –0.298 | –0.403 | –0.419 | –0.421 | 0.865^**^ | 0.210 | 0.239 | –0.572^**^ | 0.755^**^ | –0.327 | –0.530^*^ |
| *Preussia* | –0.262 | –0.483^*^ | –0.504^*^ | –0.519^*^ | 0.692^**^ | 0.296 | 0.333 | –0.358 | 0.515^*^ | –0.393 | –0.621^**^ |
| Thelebolales_Unclassified | 0.240 | –0.725^**^ | –0.684^**^ | –0.848^**^ | 0.757^**^ | 0.507^*^ | 0.162 | –0.493^*^ | 0.807^**^ | –0.758^**^ | –0.449^*^ |
| *Phaeomycocentrospora* | 0.377 | 0.413 | 0.215 | 0.585^**^ | –0.855^**^ | 0.034 | –0.332 | 0.547^*^ | –0.641^**^ | 0.486^*^ | 0.864^**^ |
| Lasiosphaeriaceae_Unclassified | –0.064 | –0.581^**^ | –0.434^*^ | –0.750^**^ | 0.797^**^ | 0.149 | 0.451^*^ | –0.435^*^ | 0.600^**^ | –0.482^*^ | –0.598^**^ |
| *Conocybe* | –0.206 | 0.507^*^ | 0.614^**^ | 0.260 | –0.409 | –0.419 | –0.266 | 0.377 | –0.319 | 0.257 | 0.170 |
| *Leptosphaeria* | –0.281 | 0.146 | 0.063 | 0.069 | 0.042 | 0.142 | –0.250 | 0.158 | 0.263 | 0.119 | 0.119 |
| *Haematonectria* | 0.174 | 0.654^**^ | 0.408 | 0.773^**^ | –0.922^**^ | –0.162 | –0.188 | 0.500^*^ | –0.783^**^ | 0.756^**^ | 0.886^**^ |
| *Nectria* | 0.333 | –0.007 | –0.017 | 0.250 | –0.383 | –0.003 | –0.332 | –0.130 | –0.172 | 0.014 | 0.351 |
| Eurotiomycetes_Unclassified | 0.026 | 0.309 | 0.155 | 0.218 | –0.535^*^ | 0.157 | 0.123 | 0.345 | –0.479^*^ | 0.208 | 0.299 |
| *Schizothecium* | –0.014 | –0.356 | –0.347 | –0.685^**^ | 0.494^*^ | 0.212 | 0.260 | –0.188 | 0.394 | –0.372 | –0.314 |
| *Paecilomyces* | 0.124 | 0.549^**^ | 0.140 | 0.667^**^ | –0.799^**^ | 0.131 | –0.235 | 0.490^*^ | –0.548^*^ | 0.630^**^ | 0.839^**^ |
| *Tetracladium* | 0.352 | –0.185 | –0.585^**^ | –0.302 | –0.001 | 0.425 | 0.249 | 0.126 | –0.165 | –0.162 | –0.073 |
| *Phoma* | 0.386 | –0.403 | –.547^*^ | –0.420 | 0.102 | 0.320 | –0.090 | –0.152 | 0.004 | –0.429 | –0.097 |
| *Stachybotrys* | –0.107 | –0.270 | –0.297 | –0.370 | 0.552^**^ | –0.024 | 0.442^*^ | –0.377 | 0.212 | –0.049 | –0.380 |
| *Cercophora* | –0.054 | –0.509* | –0.356 | –0.592^**^ | 0.708^**^ | 0.061 | 0.025 | –0.593^**^ | 0.561^**^ | –0.517^*^ | –0.502^*^ |
| *Humicola* | 0.453^*^ | –0.309 | 0.000 | –0.193 | 0.004 | –0.125 | –0.058 | –0.378 | –0.104 | –0.330 | 0.011 |
| Pleosporales_Unclassified | 0.332 | 0.136 | 0.077 | 0.407 | –0.455^*^ | –0.048 | –0.252 | 0.128 | –0.440^*^ | 0.080 | 0.176 |
| Incertae sedis_Unclassified | 0.254 | –0.098 | –0.473^*^ | 0.057 | –0.260 | 0.368 | 0.222 | 0.163 | –0.305 | 0.270 | 0.429 |
| *Metarhizium* | 0.173 | –0.413 | 0.053 | –0.529^*^ | 0.285 | –0.060 | 0.157 | –0.178 | 0.324 | –0.400 | –0.198 |
| *Epicoccum* | –0.303 | 0.303 | 0.436^*^ | 0.205 | –0.324 | –0.221 | –0.032 | 0.538^*^ | –0.290 | 0.039 | –0.182 |

** Correlation is significant at *P* = 0.01 level. * Significant at *P* = 0.05 level
