# Supplementary material for: Dynamics of soil properties and fungal community structure in continuous-cropped alfalfa fields in Northeast China
Source: PeerJ. 2019 Jun 13;7:e7127. doi: 10.7717/peerj.7127 (PMC6571135; doi:10.7717/peerj.7127)
Supplement: Supplemental Information 3 [file peerj-07-7127-s003.docx]

**Table S3** Relative abundances (%) of the dominant fungal classes of all soil samples (> 0.03% at least in one treatment).

| Class | ACC1y^a^ | ACC2y | ACC6y | ACC9y | ACC12y | ACC13y | ACC35y |
| --- | --- | --- | --- | --- | --- | --- | --- |
| Sordariomycetes | 33.64±2.27c^b^ | 42.43±1.07a | 34.44±3.24c | 35.13±1.76c | 39.92±2.52ab | 33.67±2.17c | 35.84±3.95bc |
| Incertae sedis | 15.54±1.09ab | 9.44±0.58c | 9.10±1.44c | 14.27±3.27ab | 17.28±1.76a | 13.34±2.76b | 13.97±1.46ab |
| Tremellomycetes | 13.42±1.74a | 7.59±1.36b | 14.06±1.14a | 7.41±0.32b | 3.03±0.46c | 4.15±0.83c | 3.70±0.11c |
| Dothideomycetes | 8.38±0.82d | 15.46±2.39b | 19.27±3.81a | 11.77±0.63c | 7.99±1.17d | 17.49±0.11ab | 8.33±0.92d |
| Eurotiomycetes | 4.05±0.84b | 3.85±0.71b | 5.61±1.77ab | 6.99±1.54a | 7.91±1.87a | 7.79±1.55a | 6.94±1.2a |
| Leotiomycetes | 4.37±0.22c | 5.55±0.82bc | 5.21±0.75bc | 8.68±0.95a | 2.77±0.72d | 6.43±0.29b | 4.41±0.87c |
| Agaricomycetes | 1.18±0.40c | 2.93±0.25b | 0.79±0.04c | 1.20±0.39c | 3.31±0.53b | 3.17±0.88b | 4.39±0.86a |
| Pezizomycetes | 0.98±0.35bc | 0.81±0.20c | 0.54±0.28c | 3.36±0.71a | 0.59±0.2c | 0.56±0.08c | 1.56±0.35b |
| Chytridiomycetes | 0.13±0.08b | 0.30±0.19b | 0.07±0.02b | 0.30±0.04b | 0.53±0.16a | 0.29±0.03b | 0.28±0.21b |
| Wallemiomycetes | 0.05±0.01b | 0.04±0.01b | 0.03±0.01b | 0.07±0.05ab | 0.11±0.03ab | 0.09±0.06ab | 0.12±0.06a |
| Microbotryomycetes | 0.09±0.04a | 0.02±0.02b | 0.03±0.03ab | 0.03±0.03ab | 0.01±0.00b | 0.05±0.02ab | 0.07±0.06a |
| Orbiliomycetes | 0.16±0.05bc | 0.04±0.04c | 0.14±0.05bc | 0.10±0.01c | 0.22±0.06b | 0.34±0.12a | 0.05±0.03c |
| Glomeromycetes | 0.03±0.03b | 0.06±0.03ab | 0.01±0.00b | 0.05±0.05ab | 0.07±0.03ab | 0.11±0.06a | 0.03±0.02b |
| Fungi_Unclassified | 3.22±0.57ab | 4.24±1.16a | 2.05±0.48b | 4.17±0.43a | 3.20±0.28ab | 3.81±0.38a | 3.41±0.73a |
| Ascomycota_Unclassified | 13.79±3.20a | 8.65±0.99bc | 11.42±1.3ab | 7.35±0.14c | 8.55±0.82bc | 7.50±0.74c | 10.25±1.88bc |

^a^ ACC1y, ACC2y, ACC6y, ACC9y, ACC12y, ACC13y and ACC35y represent the treatments of alfalfa continuous cropping for 1, 2, 6, 9, 12, 13 and 35 years, respectively.

^b^ Different letters within the same row indicate significant difference between treatments tested by One-Way ANOVA (*P* < 0.05). Values are the means ± SE (n = 3).
