# Supplementary material for: Dynamics of soil properties and fungal community structure in continuous-cropped alfalfa fields in Northeast China
Source: PeerJ. 2019 Jun 13;7:e7127. doi: 10.7717/peerj.7127 (PMC6571135; doi:10.7717/peerj.7127)
Supplement: Supplemental Information 4 [file peerj-07-7127-s004.docx]

**Table S4** Relative abundances (%) of the dominant fungal orders of all soil samples (> 0.1% at least in one treatment)

| Order | ACC1y^a^ | | ACC2y | | ACC6y | | ACC9y | ACC12y | ACC13y | ACC35y |
| --- | --- | --- | --- | --- | --- | --- | --- | --- | --- | --- |
| Hypocreales | | 13.60±1.97e^b^ | | 19.24±1.69d | | 20.62±2.88cd | 19.65±0.21d | 26.72±1.48a | 24.53±1.56ab | 23.35±1.78bc |
| Mortierellales | | 14.71±0.54ab | | 8.98±0.58c | | 8.42±1.38c | 13.46±3.11b | 16.72±1.18a | 13.93±0.79b | 13.13±0.98b |

| Sordariales | 13.67±2.36a | 14.39±1.01a | 7.01±1.33bc | 9.70±0.97b | 6.35±0.92c | 5.47±0.35c | 7.49±2.3bc |
| --- | --- | --- | --- | --- | --- | --- | --- |
| Pleosporales | 7.30±0.20d | 14.28±2.17b | 18.24±2.56a | 11.47±0.37c | 7.38±0.73d | 17.37±1.04a | 8.01±0.72d |
| Thelebolales | 1.75±0.06cd | 2.47±0.35bc | 3.13±0.95b | 4.88±0.31a | 0.90±0.32e | 1.49±0.13de | 0.72±0.25e |
| Eurotiales | 2.12±0.48b | 2.01±0.32b | 3.08±0.85ab | 3.06±0.54ab | 3.36±0.35a | 3.65±1.08a | 3.86±0.60a |
| Tremellales | 2.81±0.25a | 2.68±0.19a | 2.00±0.33b | 3.03±0.3a | 2.58±0.39a | 2.65±0.27a | 3.03±0.46a |
| Russulales | 0.01±0.00d | 0.00±0.00d | 0.06±0.02cd | 0.01±0.00d | 0.38±0.07a | 0.25±0.08b | 0.11±0.08c |
| Incertae sedis | 1.41±0.28b | 2.65±0.85a | 1.21±0.31b | 1.86±0.3ab | 1.70±0.45b | 1.84±0.34ab | 1.86±0.32ab |
| Agaricales | 0.77±0.25bc | 0.73±0.31bc | 0.37±0.04c | 0.57±0.06bc | 2.30±0.81a | 1.27±0.21b | 1.96±0.41a |
| Microascales | 1.76±0.22ab | 1.94±0.74a | 0.38±0.13c | 1.83±0.41ab | 2.06±0.9a | 0.88±0.05bc | 1.75±0.47ab |
| Pezizales | 0.15±0.04d | 0.77±0.21c | 0.56±0.09cd | 3.35±0.70a | 0.59±0.2cd | 0.55±0.08cd | 1.56±0.36b |
| Xylariales | 0.71±0.14cd | 0.98±0.25bc | 0.51±0.20d | 0.79±0.33cd | 1.61±0.32a | 1.28±0.06ab | 1.26±0.19ab |
| Chaetothyriales | 0.44±0.05d | 0.25±0.02d | 0.23±0.02d | 0.64±0.01cd | 0.97±0.19bc | 1.89±0.44a | 1.22±0.43b |
| Cystofilobasidiales | 9.60±0.23b | 4.91±1.45c | 12.06±0.81a | 4.37±0.27c | 0.45±0.07d | 0.93±0.03d | 0.67±0.41d |
| Helotiales | 1.90±0.33bc | 2.63±0.63ab | 1.65±0.36c | 2.60±0.38ab | 1.20±0.18c | 3.30±0.60a | 1.56±0.65c |
| Capnodiales | 0.59±0.12cd | 1.47±0.15a | 0.16±0.02e | 0.82±0.25bc | 0.32±0.09de | 0.94±0.04b | 0.56±0.2cd |
| Onygenales | 0.70±0.12d | 0.88±0.11cd | 1.22±0.43bc | 1.76±0.33a | 1.62±0.31ab | 0.83±0.16cd | 0.76±0.1cd |
| Cantharellales | 0.19±0.02b | 1.80±0.31a | 0.65±0.72b | 0.24±0.05b | 0.31±0.05b | 2.08±0.48a | 0.76±0.28b |
| Kickxellales | 0.10±0.01d | 0.06±0.01d | 0.18±0.02cd | 0.38±0.03bc | 0.73±0.30a | 0.42±0.11b | 0.19±0.05cd |
| Rhizophlyctidales | 0.01±0.00b | 0.14±0.02a | 0.02±0.00b | 0.05±0.05b | 0.02±0.00b | 0.05±0.02b | 0.01±0.00b |
| Basidiobolales | 0.09±0.03abc | 0.18±0.04ab | 0.04±0.01bc | 0.15±0.01abc | 0.17±0.18ab | 0.02±0.03c | 0.23±0.03a |
| Coniochaetales | 0.07±0.02cd | 0.30±0.03a | 0.28±0.09a | 0.19±0.02b | 0.02±0.00d | 0.21±0.03b | 0.12±0.01c |
| Spizellomycetales | 0.06±0.03b | 0.22±0.12a | 0.04±0.02b | 0.18±0.01a | 0.17±0.03a | 0.23±0.02a | 0.06±0.01b |
| Olpidiales | 0.09±0.06a | 0.02±0.01b | 0.03±0.01b | 0.03±0.02b | 0.13±0.02a | 0.03±0.00b | 0.13±0.02a |

^a^ ACC1y, ACC2y, ACC6y, ACC9y, ACC12y, ACC13y and ACC35y represent the treatments of alfalfa continuous cropping for 1, 2, 6, 9, 12, 13 and 35 years, respectively.

^b^ Different letters within the same row indicate significant difference between treatments tested by One-Way ANOVA (*P* < 0.05). Values are the means ± SE (n = 3).
