# Supplementary material for: Dynamics of soil properties and fungal community structure in continuous-cropped alfalfa fields in Northeast China
Source: PeerJ. 2019 Jun 13;7:e7127. doi: 10.7717/peerj.7127 (PMC6571135; doi:10.7717/peerj.7127)
Supplement: Supplemental Information 5 [file peerj-07-7127-s005.docx]

**Table S5** Relative abundances (%) of the dominant fungal genus of all soil samples (> 0.3% at least in one treatment)

| Genus | ACC1y^a^ | ACC2y | ACC6y | ACC9y | ACC12y | ACC13y | ACC35y |
| --- | --- | --- | --- | --- | --- | --- | --- |
| *Guehomyces* | 17.19±4.27a^b^ | 4.88±1.45c | 12.04±0.80b | 4.33±0.26c | 0.42±0.09d | 0.83±0.14d | 0.64±0.39d |
| Ascomycota_Unclassified | 13.79±3.20a | 8.65±0.99c | 11.42±1.30ab | 7.31±0.06c | 8.55±0.82c | 7.50±0.74c | 9.25±0.22bc |
| *Mortierella* | 14.70±0.56ab | 8.98±0.58c | 8.42±1.38c | 13.44±3.13b | 16.74±1.17a | 13.93±0.79b | 13.13±0.98b |
| *Leptosphaerulina* | 0.59±0.28c | 1.39±0.36b | 3.11±0.51a | 0.09±0.02d | 0.34±0.12cd | 1.28±0.09b | 0.01±0.00d |
| unidentified | 6.59±1.23a | 6.63±0.60a | 2.89±0.81b | 6.30±1.05a | 6.20±0.66a | 3.45±0.47b | 2.98±0.33b |
| Nectriaceae_Unclassified | 4.00±0.64cd | 2.70±0.95d | 9.42±1.16a | 5.35±1.08bc | 6.33±0.73b | 5.79±1.18bc | 5.09±1.49bc |
| *Gibberella* | 2.81±0.57b | 2.66±0.18b | 0.95±0.04c | 2.88±0.92b | 1.21±0.21c | 1.83±0.21bc | 3.89±0.97a |
| Fungi_Unclassified | 3.22±0.57a | 3.62±0.58a | 2.05±0.48b | 4.17±0.43a | 3.20±0.28a | 3.81±0.38a | 3.41±0.73a |
| *Cryptococcus* | 2.81±0.25a | 2.68±0.19a | 2.00±0.32b | 3.03±0.30a | 2.57±0.39a | 2.65±0.27a | 3.02±0.47a |
| Chaetomiaceae_Unclassified | 5.43±0.63a | 3.84±0.69b | 1.70±0.31f | 3.38±0.11bc | 2.49±0.07de | 2.71±0.23cd | 1.79±0.43ef |
| *Chaetomium* | 2.16±0.32a | 1.98±0.58ab | 1.37±0.70bc | 0.68±0.10c | 1.22±0.44bc | 1.09±0.33c | 0.60±0.02c |
| *Doratomyces* | 1.30±0.38a | 0.02±0.01d | 0.04±0.02d | 0.26±0.09cd | 0.53±0.25bc | 0.47±0.37bcd | 0.82±0.28b |
| *Cladosporium* | 0.56±0.10b | 1.45±0.14a | 0.16±0.03b | 0.54±0.35b | 0.42±0.19b | 0.61±0.39b | 0.55±0.20b |
| *Fusarium* | 2.63±0.22c | 7.09±0.60a | 2.88±0.25c | 3.65±0.87bc | 3.67±0.53bc | 2.89±0.39c | 4.44±0.65b |
| *Penicillium* | 1.37±0.38b | 1.36±0.22b | 1.16±0.07b | 1.84±0.61b | 2.68±0.47a | 1.51±0.06b | 1.66±0.40b |
| *Alternaria* | 0.57±0.11ab | 0.89±0.34a | 0.05±0.02c | 0.14±0.01c | 0.17±0.01c | 0.37±0.24bc | 0.56±0.29ab |
| Sordariomycetes_Unclassified | 1.03±0.24c | 0.98±0.27c | 3.71±0.32a | 2.47±0.39b | 1.03±0.30c | 1.03±0.28c | 2.96±0.30b |
| Sporormiaceae_Unclassified | 0.89±0.29c | 2.62±0.12a | 2.82±0.12a | 1.53±0.22b | 0.72±0.29cd | 0.37±0.05d | 0.51±0.17d |
| *Preussia* | 1.07±0.20b | 1.60±0.21a | 1.26±0.49ab | 1.22±0.10ab | 0.54±0.14c | 1.09±0.08b | 0.58±0.04c |
| Thelebolales_Unclassified | 1.75±0.06cd | 2.46±0.34bc | 3.13±0.95b | 4.88±0.31a | 1.00±0.18de | 1.48±0.12de | 0.72±0.24e |
| *Phaeomycocentrospora* | 0.36±0.01cd | 0.03±0.04d | 0.37±0.17cd | 0.66±0.28c | 1.21±0.30b | 1.52±0.50b | 2.05±0.22a |
| Lasiosphaeriaceae_Unclassified | 1.08±0.33b | 1.85±0.22a | 0.88±0.24b | 1.74±0.11a | 0.38±0.09c | 0.38±0.17c | 0.31±0.07c |
| *Conocybe* | 0.50±0.23a | 0.07±0.00b | 0.13±0.06b | 0.13±0.08b | 0.37±0.15a | 0.12±0.02b | 0.35±0.08a |

| *Leptosphaeria* | 0.53±0.15ab | 0.20±0.01c | 0.69±0.17a | 0.33±0.04bc | 0.36±0.22bc | 0.29±0.05bc | 0.51±0.08ab |
| --- | --- | --- | --- | --- | --- | --- | --- |
| *Haematonectria* | 0.48±0.10c | 0.06±0.01d | 0.09±0.06d | 0.03±0.01d | 1.57±0.18b | 1.41±0.36b | 2.79±0.18a |
| *Nectria* | 0.26±0.05bc | 0.17±0.06c | 0.89±0.34a | 0.62±0.25abc | 1.14±0.53a | 1.13±0.26a | 0.76±0.12ab |
| Eurotiomycetes_Unclassified | 0.79±0.22ab | 0.51±0.05bc | 0.47±0.15c | 0.67±0.10abc | 0.62±0.19abc | 0.79±0.09a | 0.77±0.14ab |
| *Schizothecium* | 0.54±0.01b | 0.56±0.13b | 0.25±0.02cd | 0.74±0.04a | 0.21±0.06d | 0.22±0.07d | 0.34±0.02c |
| *Paecilomyces* | 0.56±0.07d | 0.17±0.04e | 0.84±0.09c | 0.42±0.14d | 0.89±0.04c | 1.26±0.23b | 1.65±0.20a |
| *Tetracladium* | 1.88±0.09bc | 2.44±0.61b | 1.55±0.34c | 2.43±0.26b | 1.17±0.16c | 3.23±0.60a | 1.83±0.34bc |
| *Phoma* | 0.47±0.03c | 0.82±0.12b | 0.35±0.16c | 1.17±0.16a | 0.43±0.19c | 1.14±0.27a | 0.48±0.21c |
| *Stachybotrys* | 0.48±0.05bc | 1.43±0.32a | 0.49±0.06bc | 0.70±0.20b | 0.36±0.14c | 0.38±0.08c | 0.43±0.15bc |
| *Cercophora* | 0.03±0.02bc | 0.11±0.04a | 0.07±0.03ab | 0.09±0.02a | 0.05±0.01bc | 0.02±0.01c | 0.01±0.01c |
| *Humicola* | 0.47±0.06cd | 0.66±0.04abc | 0.39±0.04d | 0.76±0.09a | 0.70±0.11ab | 0.60±0.19abc | 0.54±0.12bcd |
| Pleosporales_Unclassified | 0.48±0.09de | 0.49±0.08de | 1.02±0.04c | 0.43±0.03e | 1.38±0.30b | 2.03±0.16a | 0.76±0.22cd |
| Incertae sedis_Unclassified | 0.45±0.12c | 0.66±0.15ab | 0.51±0.05bc | 0.63±0.13abc | 0.44±0.10c | 0.74±0.09a | 0.73±0.02a |
| *Metarhizium* | 0.52±0.19b | 0.27±0.05cd | 0.11±0.02de | 0.78±0.18a | 0.39±0.18bc | 0.03±0.01e | 0.24±0.12cde |
| *Epicoccum* | 0.40±0.05a | 0.02±0.02c | 0.06±0.04c | 0.08±0.07c | 0.11±0.07c | 0.24±0.07b | 0.10±0.09c |

^a^ ACC1y, ACC2y, ACC6y, ACC9y, ACC12y, ACC13y and ACC35y represent the treatments of alfalfa continuous cropping for 1, 2, 6, 9, 12, 13 and 35 years, respectively.

^b^ Different letters within the same row indicate significant difference between treatments tested by One-Way ANOVA (*P* < 0.05). Values are the means ± SE (n = 3).
