# Supplementary material for: Dynamics of soil properties and fungal community structure in continuous-cropped alfalfa fields in Northeast China
Source: PeerJ. 2019 Jun 13;7:e7127. doi: 10.7717/peerj.7127 (PMC6571135; doi:10.7717/peerj.7127)
Supplement: Supplemental Information 6 [file peerj-07-7127-s006.docx]

**Table S6** Relative abundances (%) of different fungal functional guilds across all samples

| Guild | OTU number | ACC1y^a^ | ACC2y | ACC6y | ACC9y | ACC12y | ACC13y | ACC35y |
| --- | --- | --- | --- | --- | --- | --- | --- | --- |
| Animal Pathogen | 25 | 1.45±0.04b^b^ | 1.62±0.13b | 0.32±0.11c | 1.98±0.88b | 6.34±3.2a | 0.28±0.07c | 0.48±0.28c |
| Arbuscular Mycorrhizal | 11 | 0.04±0.03ab | 0.05±0.02ab | 0.01±0.01b | 0.05±0.05ab | 0.06±0.02ab | 0.11±0.07a | 0.03±0.02b |
| Dung Saprotroph | 16 | 0.94±0.08bc | 1.33±0.44ab | 0.57±0.22c | 0.99±0.31bc | 0.57±0.11c | 0.68±0.14c | 1.63±0.22a |
| Ectomycorrhizal | 5 | 0.02±0.04b | 0±0b | 0.01±0.02b | 0.11±0.16b | 0.31±0.02a | 0±0b | 0.05±0.01b |
| Endophyte | 7 | 0±0b | 0.04±0.05a | 0.01±0.01ab | 0±0b | 0±0b | 0±0b | 0.01±0.01ab |
| Ericoid Mycorrhizal | 2 | 0±0a | 0±0a | 0.01±0.01a | 0.01±0.00a | 0±0a | 0.04±0.06a | 0.01±0.01a |
| Fungal Parasite | 7 | 0.29±0.24b | 0.19±0.05b | 0.63±0.36b | 1.12±0.42a | 0.30±0.05b | 0.43±0.08b | 0.39±0.06b |
| Lichenized | 1 | 0.86±0.70bc | 1.39±0.35b | 2.77±0.55a | 0.29±0.21cd | 0.33±0.12cd | 1.30±0.11b | 0.03±0.03d |
| Litter Saprotroph | 4 | 0.01±0.01a | 0.01±0.01a | 0.01±0.02a | 0.01±0.01a | 0.01±0.00a | 0.01±0.01a | 0±0a |
| Plant Pathogen | 84 | 5.85±0.44c | 6.54±0.59c | 8.81±1.49b | 5.41±0.62c | 9.01±0.12b | 8.27±1.55b | 10.92±0.75a |
| Plant Saprotroph | 41 | 1.66±0.24c | 4.60±1.24ab | 4.93±0.62a | 3.43±0.89b | 1.82±0.55c | 1.54±0.12c | 1.87±0.38c |
| Soil Saprotroph | 25 | 0.40±0.08b | 0.45±0.13b | 0.45±0.13b | 0.53±0.27ab | 0.38±0.06b | 0.78±0.15a | 0.36±0.06b |
| Wood Saprotroph | 148 | 17.73±1.31bc | 19.18±1.72b | 13.69±0.69d | 14.47±0.78d | 13.24±0.35d | 25.17±3.12a | 15.67±2.27cd |
| Undefined Saprotroph | 490 | 36.25±1.51a | 37.95±1.68a | 29.47±3.28b | 36.62±0.86a | 39.31±3.09a | 36.59±2.67a | 39.55±3.04a |
| unknow | 1045 | 34.50±1.00a | 26.65±0.94b | 38.31±1.59a | 34.97±1.42a | 28.31±4.66b | 24.78±3.78b | 29.03±0.60b |

^a^ ACC1y, ACC2y, ACC6y, ACC9y, ACC12y, ACC13y and ACC35y represent the treatments of alfalfa continuous cropping for 1, 2, 6, 9, 12, 13 and 35 years, respectively.

^b^ Different letters within the same row indicate significant difference between treatments tested by One-Way ANOVA (*P* < 0.05). Values are the means ± SE (n = 3).
