# Supplementary material for: Dynamics of soil properties and fungal community structure in continuous-cropped alfalfa fields in Northeast China
Source: PeerJ. 2019 Jun 13;7:e7127. doi: 10.7717/peerj.7127 (PMC6571135; doi:10.7717/peerj.7127)
Supplement: Supplemental Information 7 [file peerj-07-7127-s007.docx]

**Table S7** Relative abundance (%) and taxa information of dominant OTUs belonged to plant pathogen (a) and their correlations with soil environmental variables (b)

(a)

| OTU ID | ACC1y^a^ | ACC2y | ACC6y | ACC9y | ACC12y | ACC13y | ACC35y | Taxonomy |
| --- | --- | --- | --- | --- | --- | --- | --- | --- |
| OTU1786 | 2.07±0.31a^b^ | 2.06±0.19a | 0.64±0.10b | 1.79±0.30a | 1.10±0.29b | 0.90±0.07b | 2.17±0.41a | Ascomycota-Sordariomycetes-*Fusarium equiseti* |
| OTU1176 | 0.64±0.07b | 0.52±0.02b | 0.52±0.09b | 0.63±0.12b | 0.34±0.07b | 0.95±0.18a | 1.05±0.33a | Ascomycota-Sordariomycetes-*Fusarium incarnatum* |
| OTU211 | 0.48±0.10c | 0.07±0.01cd | 0.05±0.03cd | 0.02±0.01d | 1.14±0.50b | 1.30±0.30b | 2.62±0.17a | Ascomycota-Sordariomycetes-*Haematonectria haematococca* |
| OTU1880 | 0.05±0.01c | 0.82±0.12ab | 0.17±0.03c | 0.78±0.20ab | 0.68±0.22ab | 0.88±0.32a | 0.50±0.07b | Ascomycota-Sordariomycetes-*Lectera longa* |
| OTU1028 | 0.17±0.06e | 0.62±0.11a | 0.35±0.02cd | 0.22±0.03de | 0.55±0.07ab | 0.42±0.08bc | 0.64±0.12a | Ascomycota-Sordariomycetes-*Cylindrocarpon* sp |
| OTU1311 | 0.10±0.03c | 0.02±0.01c | 0.02±0.02c | 0.01±0.01c | 0.64±0.06a | 0.63±0.10a | 0.25±0.04b | Ascomycota-Eurotiomycetes-*Cyphellophora* sp |

^a^ ACC1y, ACC2y, ACC6y, ACC9y, ACC12y, ACC13y and ACC35y represent the treatments of alfalfa continuous cropping for 1, 2, 6, 9, 12, 13 and 35 years, respectively.

^b^ Different letters within the same row indicate significant difference between treatments tested by One-Way ANOVA (*P* < 0.05). Values are the means ± SE (n = 3).

(b)

| OTU ID | pH | Moisture | TC | TN | TP | TK | NH_4_^+^–N | NO_3_^–^–N | AP | AK | Year |
| --- | --- | --- | --- | --- | --- | --- | --- | --- | --- | --- | --- |
| OTU1786 | –0.204 | 0.146 | 0.128 | –0.041 | –0.082 | –0.150 | 0.211 | 0.349 | –0.196 | 0.287 | 0.160 |
| OTU1176 | 0.026 | 0.366 | –0.154 | 0.357 | –0.546^*^ | 0.367 | –0.022 | 0.494^*^ | –0.412 | 0.519^*^ | 0.600^**^ |
| OTU211 | 0.122 | 0.682^**^ | 0.375 | 0.750^**^ | –0.898^**^ | –0.102 | –0.241 | 0.533^*^ | –0.761^**^ | 0.756^**^ | 0.893^**^ |
| OTU1880 | 0.460^*^ | –0.190 | –0.204 | –0.017 | –0.141 | 0.037 | –0.085 | –0.287 | –0.258 | –0.118 | 0.142 |
| OTU1028 | 0.197 | 0.333 | 0.159 | 0.506^*^ | –0.321 | –0.256 | 0.160 | 0.036 | –0.507^*^ | 0.603^**^ | 0.319 |
| OTU1311 | 0.396 | 0.274 | 0.448^*^ | 0.583^**^ | –0.712^**^ | –0.317 | –0.264 | 0.268 | –0.707^**^ | 0.204 | 0.497^*^ |

TC, TN, TP, TK, AP and AK indicate soil total carbon, total nitrogen, total phosphorus, total potassium, available phosphorus and available potassium, respectively.

** Correlation is significant at *P* = 0.01 level. * Significant at *P* = 0.05 level.
