# Supplementary material for: Dynamics of soil properties and fungal community structure in continuous-cropped alfalfa fields in Northeast China
Source: PeerJ. 2019 Jun 13;7:e7127. doi: 10.7717/peerj.7127 (PMC6571135; doi:10.7717/peerj.7127)
Supplement: Supplemental Information 8 [file peerj-07-7127-s008.docx]

**Table S8** Mantel test results for the correlation between fungal community composition and environmental variables.

| Variable^a^ | r | *P* |
| --- | --- | --- |
| pH | **0.175**^b^ | 0.020 |
| Mositure | –0.093 | 0.859 |
| TC | 0.108 | 0.125 |
| TN | 0.083 | 0.184 |
| TP | **0.463** | 0.001 |
| TK | 0.057 | 0.252 |
| NH_4_^+^-N | 0.006 | 0.461 |
| NO_3_^-^-N | –0.015 | 0.550 |
| AP | **0.399** | 0.001 |
| AK | –0.036 | 0.642 |

^a^ TC, TN, TP and TK indicate soil total carbon, total nitrogen, total phosphorus and total potassium, respectively; AP and AK indicate soil available phosphorus and available potassium, respectively; NH_4_^+^-N, ammonium nitrogen, NO_3_^-^-N, nitrate nitrogen.

^b^ Values in bold indicate significant correlation (*P* < 0.05).
